# Multi-session Transcranial Direct Current Stimulation Over Primary Motor Cortex Facilitates Sequence Learning, Chunking, and One Year Retention

**DOI:** 10.3389/fnhum.2020.00075

**Published:** 2020-03-12

**Authors:** Brian Greeley, Jonathan S. Barnhoorn, Willem B. Verwey, Rachael D. Seidler

**Affiliations:** ^1^School of Kinesiology, University of Michigan, Ann Arbor, MI, United States; ^2^Department of Psychology, University of Michigan, Ann Arbor, MI, United States; ^3^Department of Cognitive Psychology and Ergonomics, University of Twente, Enschede, Netherlands; ^4^Department of Applied Physiology and Kinesiology, University of Florida, Gainesville, FL, United States

**Keywords:** tDCS, motor sequence learning, chunking, primary motor cortex (M1), prefrontal cortex (PFC)

## Abstract

Transcranial direct current stimulation (tDCS) over the primary motor cortex (M1) can facilitate motor learning, but it has not been established how stimulation to other brain regions impacts online and offline motor sequence learning, as well as long-term retention. Here, we completed three experiments comparing the effects of tDCS and sham stimulation to the prefrontal cortex (PFC), M1, and the supplementary motor area complex to understand the contributions of these brain regions to motor sequence learning. In Experiment 1, we found that both left and right PFC tDCS groups displayed a slowing in learning in both reaction time and number of chunks, whereas stimulation over M1 improved both metrics over the course of three sessions. To better understand the sequence learning impairment of left PFC anodal stimulation, we tested a left PFC cathodal tDCS group in Experiment 2. The cathodal group demonstrated learning impairments similar to the left PFC anodal stimulation group. In Experiment 3, a subset of participants from the left PFC, M1, and sham tDCS groups of Experiment 1 returned to complete a single session without tDCS on the same sequences assigned to them 1 year previously. We found that the M1 tDCS group reduced reaction time at a faster rate relative to the sham and left PFC groups, demonstrating faster relearning after a one-year delay. Thus, our findings suggest that, regardless of the polarity of stimulation, tDCS to PFC impairs sequence learning, whereas stimulation to M1 facilitates learning and relearning, especially in terms of chunk formation.

## Introduction

According to the Cognitive framework for Sequential Motor Behavior (C-SMB), sequence learning depends on communication between a central and a motor processor (Verwey et al., 2015). The central processor loads individual movements early in learning, motor chunks later in learning, and it orchestrates the transition of motor sequences from short- to long-term memory. The motor processor is responsible for executing individual responses early in learning and entire motor chunks after extended practice (Abrahamse et al., 2013; Verwey et al., 2015). The authors of the *C-SMB* framework posit that learning in the discrete sequence production (*DSP*) task (see Abrahamse et al., 2013) involves three separate modes: a reaction mode, occurring early in learning when participants are responding to each individual stimulus, a central-symbolic mode in which sequence execution relies on verbal and/or spatial sequence representations, and a chunking mode occurring later in learning when execution is based on motor chunk representations. The prefrontal cortex (PFC) is assumed to prepare the neural system for sequence execution and would determine the contributions of each of the sequence execution modes. Bilateral dorsal premotor cortex, bilateral posterior parietal cortex, precuneus, and preSMA are thought to be involved in the reaction mode (Verwey et al., 2019). The premotor cortex and bilateral posterior parietal and/or temporal areas are involved in the central-symbolic mode. The posterior striatum and the SMA play key roles in representing motor chunks in the chunking mode by controlling primary motor cortex (M1), but with extensive practice, M1 may exclusively represent the motor chunks (Karni et al., 1998; Verwey et al., 2002; Abrahamse et al., 2013).

Neuroimaging studies provide the support that the prefrontal cortices play a role in sequence learning including chunk segmentation and aiding in the transfer of sequence knowledge from short- to long-term memory. For example, fMRI studies have shown that prefrontal regions are active when subjects explicitly learn a sequence following the serial reaction time task (Hazeltine et al., 1997; Honda et al., 1998; Willingham et al., 2002) as well as when they practice probabilistic sequences under explicit and implicit conditions (Aizenstein et al., 2004; Yang and Li, 2012). There are at least two other fMRI studies that support the role of the dorsolateral prefrontal cortices (DLPFC) in chunk segmentation (Pammi et al., 2012; Wymbs et al., 2012). Moreover, the prefrontal cortices were found to interact with the medial temporal lobe during both encoding and retrieval of motor sequences, facilitating long-term memory (Simons and Spiers, 2003). Thus, many neuroimaging studies have implicated a role for the PFC in sequence learning, perhaps especially focusing on their higher-level control.

Non-invasive brain stimulation studies have helped solidify the role of the prefrontal cortices in sequence learning and consolidation. For example, TMS disrupting the DLPFC while participants completed the serial reaction time task resulted in impaired procedural learning (Pascual-Leone et al., 1996). In another study, the application of excitatory (anodal) Transcranial direct current stimulation (tDCS) over the right DLPFC during a probabilistic sequence learning task resulted in enhanced retention (Janacsek et al., 2015). Together, these findings suggest that the prefrontal cortices facilitate consolidation, potentially through mechanisms similar to long-term potentiation (Islam et al., 1995, 1997). Thus, brain stimulation studies provide further support for the *C-SMB* framework in that the prefrontal cortices contribute to sequence learning.

It is unclear though whether there is a prefrontal hemispheric specialization in sequence learning. Wilkinson et al. (2010) used TMS to inhibit participants’ left PFC during the acquisition of an implicit probabilistic sequence and observed no effect on performance. These results suggest that the left PFC has no role in sequence learning; however, the authors did not include a right PFC group, complicating interpretations. Another study using excitatory (anodal) tDCS over either the left or the right PFC found no effect on probabilistic sequence learning, but the right PFC stimulation group did show a sequence consolidation benefit (Janacsek et al., 2015). In contrast, we recently reported that anodal tDCS over left but not the right PFC improved probabilistic sequence learning, but only in participants who remained implicit about the sequence (Greeley and Seidler, 2019). Additionally, we found no beneficial effect of anodal tDCS either over the right or left PFC when participants became explicitly aware of the sequence. These results suggest that the exact role of the right or left PFC in sequence learning may be time—(acquisition vs. consolidation) and/or task—(implicit vs. explicit; centrally presented vs. spatially cued responses) dependent.

In contrast, a few studies suggest that prefrontal cortical activity can also interfere with aspects of sequence learning. Galea et al. (2010) disrupted either the left or right DLPFC with theta-burst TMS immediately *after* participants learned a sequence in the serial RT task and found enhanced retention. Likewise, attending to the execution of an automated skill, assumed to increase the involvement of the prefrontal cortices, results in poorer performance (Beilock and Carr, 2001; Beilock et al., 2002; Gray, 2004). These studies suggest that engaging attention or activating the PFC can interfere with the retention or execution of highly practiced sequences. Therefore, disrupting the prefrontal cortices might facilitate learning. An example of this comes from a study by Zhu et al. (2015) where cathodal tDCS over left PFC resulted in an *advantage* for golf putting performance relative to a sham group. In summary, this subset of studies suggests that prefrontal cortical activity may in fact interfere with execution or retention of practiced sequences.

There is extensive evidence for the role of M1 in sequence learning. For example, Karni et al. (1998) reported fMRI BOLD fluctuations in the left M1 in the first minutes of explicit motor sequence learning as well as during sequence production 8 weeks later when compared to an unlearned sequence, suggesting a role for M1 in both online and offline learning. Nitsche et al. (2003) found that anodal tDCS to M1 facilitated motor learning in the serial reaction time task within a single session (i.e., online gains). Stimulation over M1 while learning an isometric pinch force sequence task over the course of 5 days showed greater motor skill learning that was driven by offline effects, and this remained evident 3 months later (Reis et al., 2009). These findings are compatible with the *C-SMB* framework and other models (Doyon and Benali, 2005; Verwey et al., 2019) in assuming the role of the motor cortex in motor sequence execution.

There is also evidence that the preSMA is involved in learning new action sequences and chunking together individual actions. The preSMA is involved in the acquisition of new action sequences in non-human primates (Nakamura et al., 1998, 1999) as well as in humans (Grafton et al., 1995; Willingham et al., 2002; Verwey et al., 2019). Consistent with the *C-SMB* framework, previous studies suggest the preSMA is also involved in chunking, specifically motor chunk loading prior to movement execution. Using TMS, two studies have disrupted the preSMA while participants produced an overlearned sequence (Kennerley et al., 2003; Ruitenberg et al., 2014). The disruption led to slower reaction times at a chunk point suggesting the preSMA is involved in initiating a new action sequence and loading in individual motor chunks.

In the current study, we sought to adjudicate the two competing views regarding prefrontal cortical contributions to sequence learning, specifically whether excitatory tDCS to either the left or the right PFC would facilitate or interfere with sequence learning over the course of 3 days. In Experiment 1, we tested four different anodal tDCS groups in which either the left PFC, the right PFC, the left M1, or the SMA complex were stimulated, compared with a sham condition, to understand how excitatory stimulation affects motor learning over the course of three sessions. In Experiment 2, we tested a left PFC cathodal tDCS group performing the same task. If engaging prefrontal regions results in the facilitation of learning, one could expect that left PFC cathodal tDCS would interfere and decrease reaction times and chunk formation relative to the sham group. In Experiment 3, we assessed long-term retention differences in a subset of participants from the sham, left PFC, and M1 tDCS groups of Experiment 1. We hypothesized that anodal stimulation to the left PFC and M1 during the practice of the sequences would facilitate the long-term memory of these sequences, evaluated 1 year later.

## Experiment 1

In the first experiment, groups of participants received anodal tDCS over either left M1, left PFC, right PFC, SMA complex, or no stimulation (sham protocol) while they practiced one simple and one complex six-item sequence in the *DSP* task. Given earlier findings (Karni et al., 1998; Reis et al., 2009), we hypothesized that M1 stimulation would facilitate learning as evidenced by online and offline gains in response time. We also anticipated that stimulation to M1 would reduce the number of motor chunks after extended practice. Based on previous non-invasive brain stimulation studies we further hypothesized that stimulating either the left (Greeley and Seidler, 2019) or right PFC (Janacsek et al., 2015) would facilitate online sequence learning as well as consolidation. Alternatively, stimulation to the PFC may result in reduced learning (Galea et al., 2010) and chunking, potentially due to a reduction in motor chunk contribution (Verwey et al., 2015). Finally, we anticipated that applying anodal stimulation to the preSMA would result in enhanced learning as evidenced by shorter reaction times and a lower number of chunks relative (Kennerley et al., 2003) to the sham group (i.e., more items per chunk).

## Method

### Participants

Sixty-five young adult participants (mean age 20.5 ± 2.4, 27 males) were recruited from the University of Michigan campus and greater Ann Arbor area. All participants were right-handed and reported having no history of mental health events, drug abuse, or psychiatric disorders. During the first session, all participants signed a consent form approved by the University of Michigan Institutional Review Board, verbally answered an alcohol and drug abuse questionnaire, completed the Beck Depression Inventory (Beck et al., 1996), a custom tDCS screening form, and the Montreal Cognitive Assessment (Nasreddine et al., 2005). All participants scored ≥23 on the MOCA and scored <13 on the Beck Depression Inventory.

### Discrete Sequence Production (DSP) Task

The *DSP* task used for this study was a modified version of that used by Ruitenberg et al. (2014) programmed in E-Prime (version 2.0). Each participant was randomly assigned two, six-item sequences for the duration of the study. One of the sequence pairs was considered simple and had an imposed structure (one of cvncvn, vbcvbc, ncbncb, and bnvbnv), whereas the other sequence was complex and did not have an imposed structure (one of nvbcbv, cbnvnb, vncbcn, bcvnvc). In order to investigate the role of the central processor and of the prefrontal cortices in sequence learning and to simplify data presentation, we limited our analyses to the complex sequences. Participants placed the index, middle, ring, and pinky fingers of their right hand on the C, V, B, and N keys of a keyboard, respectively. Four 2.8 × 2.8 cm horizontally aligned white squares with black trim were presented in the middle of a computer monitor with a white background; the squares were 1.4 cm apart. The blank squares were randomly presented for either 500 or 1,000 ms before the first stimulus was displayed. As soon as one of the squares was filled in by a light green color (for up to 2,000 ms), participants were told to make a response with the spatially corresponding key. Once a correct response was given, the green square returned to white and then the next square in the sequence would turn light green. Once all six squares of the sequence were successfully responded to, the display turned to white for either 500 or 1,000 ms. If participants made an incorrect key press, the message “mistake, again” was displayed in red at the bottom of the screen for 1,000 ms. If a participant did not respond within the 2,000 ms window, the message, “no response, again” was displayed in red at the bottom of the screen for 1,000 ms. Participants practiced each of their two sequences eight times during each block of practice. The two sequence types (simple and complex) were presented in a random fashion. If a participant made an error either by pressing the incorrect key or not responding to the stimulus at all during the first trial, the sequence was not replaced.

Halfway through a block (after eight trials), participants observed a feedback screen for 10 s which displayed error percentage, mean reaction time, and a numerical countdown starting from 10. Once the numerical countdown reached zero, participants immediately started the next practice trials. At the end of a block, participants observed another feedback screen for 50 s. The feedback screen had the same information as to when it was presented during a sub-block, and additionally, text at the bottom of the screen that read, “After this, practice block × will start.”

Before blocks 2, 3, 4, 5, and 6 during sessions one and two, immediately following the feedback screen after a block, participants observed another screen that read, “As you have noticed, there are two fixed sequences. Please learn them! We will continue with the same task.” Thus, participants were able to practice their assigned sequences a total of 96 times for each sequence during session one.

During sessions two and three immediately following the *DSP* questionnaire (see below), participants performed the test phase of the *DSP* task. It consisted of four conditions, each comprised 48 trials (24 trials of each sequence) and followed the same structure as practice. In a *familiar* condition, the stimuli were presented in the same way as practice. In the *single-stimulus* condition, participants performed their practiced sequences; however, only the first square of the sequence turned green. After the participant pressed the correct key of the first green square, the squares remained white, and participants completed the rest of the sequence (five key presses) without the squares turning green. In the *mixed-familiar* condition, 75% of the trials had changes to the sequences such that two of the six stimuli were changed whereas in 25% of the trials the sequences were the same as practice. In the fourth condition, which we called *unfamiliar*, there were two sequences of the above set that the participant had not previously experienced.

### tDCS Setup

Participants were randomly assigned to one of five tDCS groups for the duration of the study. Four of the five tDCS groups received anodal stimulation, whereas the fifth group received sham stimulation. The electrode placement was determined using the 10-20 EEG system. For right and left prefrontal stimulation groups, the anode was either placed over scalp location F4 or F3 and the cathode over the contralateral orbit. For the left M1 stimulation group, the anode was placed over the scalp location C3 and the cathode over the contralateral orbit. To determine electrode placement for the SMA complex stimulation group, we used the peak 3D coordinates for the preSMA (Mayka et al., 2006). Then, we used the locations of the international 10–20 cortical projection and head surface points to calculate the optimal electrode location (Okamoto et al., 2004). Based on this method, we took 8.7% of the measured distance between the nasion and inion and placed the anode anterior to that distance from Cz with the cathode over Fpz. This distance was approximately 3 cm anterior to Cz and 1 cm anterior to other tDCS studies targeting similar brain regions (Hayduk-Costa et al., 2013; Carter et al., 2015). Given the proximity of preSMA and SMA, we refer to this stimulation as targeting the SMA complex for the duration of this article. The sham stimulation group received the same montage as the real, M1 tDCS group. Stimulation current was 2 mA and was administered using a conventional tDCS device (Soterix Medical Inc., New York, NY, USA) for a maximum of 20 min *via* two rubber electrodes which were placed inside two saline-soaked sponges. For the sham group, the current ramped up to 2 mA, then immediately ramped back down over a period of 30 s. The anode electrode size was always 5 × 5 cm and the cathode was 5 × 5 cm except for the SMA complex group, where the cathode was 5 × 7 cm, as previous literature has demonstrated this to be an effective size for stimulating the SMA complex (Vollmann et al., 2013; Table 1). tDCS setup was identical during sessions one and two, and tDCS was not administered during session three.

**Table 1:** Summary of transcranial direct current stimulation (tDCS) conditions and results for each experiment.

	Group name	Anode location	Cathode location	Electrode size (cm)	Current intensity	Duration (mins)	Behavioral outcome
Experiment 1 excitatory tDCS	Right PFC	F4	Contralateral orbit	5 × 5	2 mA	20	+ RT
							+ chunks
	Left PFC	F3	Contralateral orbit	5 × 5	2 mA	20	+ RT
							+ chunks
	Left M1	C3	Contralateral orbit	5 × 5	2 mA	20	− RT
							− chunks
	SMA complex	3 cm anterior to Cz	Fpz	5 × 5 (anode)	2 mA	20	− RT
							+ − chunks
				5 × 7 (cathode)
	Sham	C3	Contralateral orbit	5 × 5	2 mA	0.5	N/A
Experiment 2 inhibitory tDCS	Left PFC	Contralateral orbit	F3	5 × 5	2 mA	20	+ RT
							+ chunks
Experiment 3 follow-up	Left PFC	N/A	N/A	N/A	N/A	N/A	Less Forgetting
	Left M1	N/A	N/A	N/A	N/A	N/A	Faster relearning
	Sham	N/A	N/A	N/A	N/A	N/A	N/A

*A plus sign (+) preceding “RT” or “chunks” in the behavioral outcome column signifies longer reaction time (RT) or a greater number of chunks relative to sham, respectively. In contrast, a negative sign (−) signifies shorter reaction time or fewer chunks relative to sham*.

### ROAST Model

**Figure 1:**
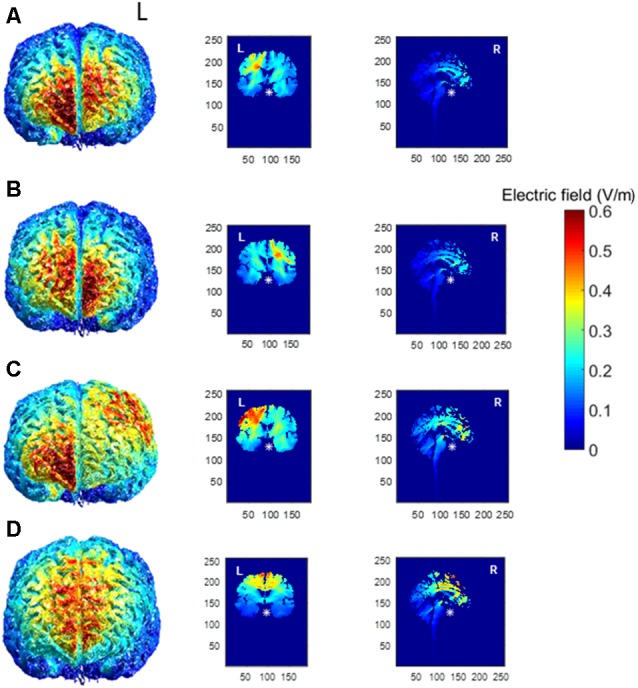
Electric field magnitude distribution in the whole brain (anterior), coronal, and sagittal slices for the **(A)** left prefrontal montage, **(B)** right prefrontal montage, **(C)** the left primary motor cortex (M1) montage, and **(D)** for the SMA complex montage. The left hemisphere is denoted by L, whereas the right hemisphere is denoted by R.

Realistic volumetric-Approach to Simulate Transcranial Electric Stimulation, or ROAST (version 1.0), is an open-source automated Matlab script available for modeling the presumed current produced by transcranial electric stimulation (Huang et al., 2019). We ran the model a total of four times to account for the four different tDCS electrode montages used in this experiment (right PFC, left PFC, M1, SMA complex) to better understand the current density. The output of the model can be observed for the left PFC anode, right orbitofrontal cortex cathode montage (Figure 1A), the right PFC anode, left orbitofrontal cortex cathode montage (Figure 1B), the left M1 anode, right orbitofrontal cortex cathode montage (Figure 1C), and the SMA complex anode, orbit cathode montage (Figure 1D). We customized the input parameters of the model for the right PFC, left PFC, and M1 and changed the height of the electrode to 1 mm, the sponge height to 2 mm, and the radius of the electrodes to 3.56 as we used two 5 × 5 cm electrodes and not a high-definition tDCS system. Currently, the model does not allow input for two different sized electrodes, which we used for the SMA complex montage (we used a 5 × 5 cm electrode for the anode and a 5 × 7 cm electrode for the cathode). Also, the input for the electrodes in ROAST was limited to the 10-10 EEG system, however, we determined the site of the anode for the SMA complex montage as anterior to Cz by 8.7% of the distance between the nasion and the inion. Thus, output for the SMA complex is not an entirely accurate representation and should be interpreted with caution.

### Procedure

As this study is part of a larger aging study, participants completed a series of neuropsychological assessments during session one. Assessments included Thurstone’s card rotation task (two-dimensional mental rotation), a custom computerized version of a visual search task, the digit symbol substitution task (Wechsler, 1958), a modified version of the visual array change working memory assessment (Luck and Vogel, 1997; Bo et al., 2009), and then three trials of the Purdue pegboard task (Tiffin and Asher, 1948). Lastly, we measured the participant’s grip strength. Here, we do not report analyses on these neuropsychological assessments and instead focus on the sequencing data. We had participants take a mandatory 3–5 min break before we began tDCS set-up. After tDCS set-up, we turned on the stimulation to 1 mA for 15 s (pre-stimulation tickle) to ensure satisfactory contact quality. After this brief stimulation period, participants completed a shortened 10-item PANAS mood inventory, then the experimenter explained the instructions for the *DSP* task. We then started the tDCS stimulation and let it ramp up to full intensity (always 2 mA) and asked whether participants were comfortable (including sham participants). Once participants confirmed they were comfortable, we started the *DSP* task. After six blocks of practice in the *DSP* task (96 repetitions of each sequence), tDCS was turned off. If the task was not completed by 20 min of tDCS, stimulation was stopped at that time. Then, we administered a second version of the digit symbol task, the custom mood survey, and a custom tDCS side effects questionnaire. After the participants completed the tDCS questionnaire, we removed the electrodes, and sent the participants home with a physical exercise questionnaire as well as the Edinburgh handedness questionnaire (Oldfield, 1971). Session one lasted approximately 2 h and 30 min.

During session two, separated by at least one night’s sleep but no longer than 72 h after session one, participants completed the card rotation task, followed by the digit symbol substitution task. tDCS was then set-up and the pre-stimulation tickle was administered. We then administered the mood survey and summarized the instructions of the *DSP* task. Once tDCS reached full intensity and the participant was comfortable, they completed six blocks of *DSP* practice (blocks 7–12, totaling 192 practice trials per sequence at this point in the study). After practice, the *DSP* questionnaire was administered (see below; tDCS stimulation was off at this point), followed by instructions of the test portion of the *DSP* task (see below). Once participants understood the test portion of the *DSP* task and had completed all four conditions, participants completed the digit symbol substitution coding task again, the mood survey, and the tDCS side effects questionnaire.

On the third session, separated by at least one night’s sleep but no longer than 72 h after session two, participants completed two blocks of practice on the *DSP* task (blocks 13–14, totaling 224 practice trials per sequence at this point in the study), followed by the *DSP* questionnaire, which was followed by the test phase. Participants were offered a break, then completed the card rotation test, the visual search task, the digit symbol substitution coding task, and the visual array change task. Afterward, participants completed an exit survey, which asked whether they thought they had been in the sham or real tDCS group.

### DSP Questionnaire

At the end of practice for sessions two and three, we administered a questionnaire probing participants’ awareness of the sequences. The first three questions tested the participants’ knowledge of the sequences by having them write down, verbally tell the experimenter, and choose from a list of 18 possible sequences they practiced, respectively. Participants were also asked about strategies used to remember sequences, and whether they realized there were two fixed sequences (for a review of similar awareness results in the DSP sequences, see Verwey et al., 2016).

### Data Analyses

Our primary outcomes for this study were reaction time, number of (motor) chunks, and number of errors for the complex sequences. However, in order to understand the effect of tDCS on chunking, we also conducted an additional supplementary analysis limited to the number of chunks for the simple sequences (see Supplementary Material). We implemented a linear mixed model using the software Stata for reaction time and number of chunks using trials as a continuous factor and stimulation group, and session as a blocked factor. We chose a linear mixed model because we wanted to investigate the *rate* of learning for each stimulation group as every participant had a different number of trials due to the removal of errors, an issue that disappears when using a linear mixed model. Additionally, linear mixed model is a sensitive measure of learning as it does not require trials to be averaged over blocks. In the mixed model, we used random intercepts and fixed slopes for each participant, as using random slopes for each participant did not change the results. To identify the number of chunks for each keypress, we used a computational model developed by Acuna et al. (2014), which uses reaction times as well as the covariation across key presses in order to detect chunk boundaries. To compute effect sizes for each linear mixed model, we used the *F* statistic and *df* from each model to calculate eta-squared (*η*^2^). For the number of errors, we used Friedman’s test followed by a series of Wilcoxon follow-ups. We also used two, two-way ANOVAs with four contrasts (right PFC vs. sham, left PFC vs. sham, etc.) to investigate offline learning gains as well as overall differences in reaction time and number of chunks. For the ANOVA contrasts, we normalized each trial’s reaction time or number of chunks to the first trial. We included the additional ANOVA contrasts to assess overall differences between the stimulation groups, whereas the linear mixed model assessed differences in the *rate* of learning. Offline learning gains were calculated by subtracting the mean of one complete sequence (e.g., six key presses) from the first trial of a session from the mean of six key presses from the last trial within a session (e.g., mean RT trial 192—session-one—mean RT trial 193 session-two). Beta and standard error values are presented relative to sham. We did not correct for multiple comparisons (Rothman, 1990).

## Results and Discussion

### Reaction Time

Across the five tDCS stimulation groups, the linear mixed model revealed that reaction time changed faster across trials in the first session than in the second (*β* = −0.88, *SE* = 0.02, *p* < 0.001). Reaction time across trials in session three changed significantly faster relative to the trials in session two (*β* = −0.38, *SE* = 0.08, *p* < 0.001).

**Figure 2:**
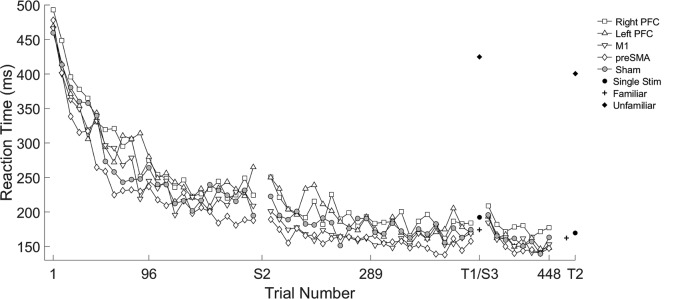
Mean reaction time (ms) of the complex sequence as a function of trial number over the course of learning with mean reaction time for the testing conditions. Displayed means were binned across every eight trials. S2, S3, T1, and T2 on the x-axis represent the start of session two, session three, and testing conditions one and two, respectively.

We found several significant differences in the slopes of reaction time across trials within-session by the tDCS stimulation group. In the first session, the left PFC group reduced reaction time more slowly across trials relative to the sham group (*β* = 0.10, *SE* = 0.05, *p* = 0.037). In the second session, stimulation to left PFC resulted in a significantly faster rate of change in reaction time across trials relative to the sham group (*β* = −0.19, *SE* = 0.05, *p* < 0.001; Figure 2; Table 2). There were no significant interactions in the third session. Thus, stimulation to left PFC slowed sequence production during session one, which resulted in a “catch-up” effect during session two. There were no other significant differences.

**Table 2:** The output from the linear mixed model across Experiments 1, 2, and 3.

Study	Interaction	Data Type	β	Std. Error	*P*	*η*^2^
Experiment 1 excitatory tDCS	Session × Trial					0.05
	Session 1 vs. Session 2	RT	−0.88	0.02	<0.001
	Session 3 vs. Session 2	RT	−0.38	0.08	<0.001
	Session × Stimulation × Trial					<0.01
	Session 1 × L PFC vs	RT	0.10	0.05	0.037
	Session 1 × Sham				
	Session 2 × L PFC vs	RT	−0.19	0.05	<0.001
	Session 2 × Sham				
	Session × Trial					<0.01
	Session 1 vs. Session 2	Chunk	<−0.01	<0.01	<0.001
	Session 3 vs. Session 2	Chunk	<−0.01	<0.01	0.001
	Stimulation × Trials					<0.01
	SMA complex vs. Sham	Chunk	<−0.01	<0.01	<0.001
	Right PFC vs. Sham	Chunk	<−0.01	<0.01	<0.001
	Session × Stimulation × Trial					0.01
	Session 1 × L PFC vs	Chunk	< 0.01	<0.01	<0.001	
	Session 1 × Sham				
	Session 2 × R PFC vs	Chunk	< -0.01	<0.01	<0.001
	Session 2 × Sham				
	Session 2 × L PFC vs	Chunk	< -0.01	<0.01	<0.001
	Session 2 × Sham				
	Session 1 × M1 vs	Chunk	<−0.01	<0.01	<0.001
	Session 1 × Sham				
	Session 1 × SMA complex vs	Chunk	<−0.01	<0.01	<0.001
	Session 1 × Sham				
	Session 3 × SMA complex vs	Chunk	−0.02	<0.01	<0.001
	Session 3 × Sham				
	Session 3 × R PFC vs	Chunk	−0.01	<0.01	0.001
	Session 3 × Sham					
Experiment 2 inhibitory tDCS	Session × Trial					0.04
	Session 1 vs. Session 2	RT	−0.82	0.03	<0.001
	Session 3 vs. Session 2	RT	−0.31	0.11	0.004
	Session × Stimulation × Trial					<0.01
	Session 1 × anodal L PFC vs	RT	0.16	0.05	0.001
	Session 1 Sham				
	Session 2 × anodal L PFC vs	RT	−0.21	0.05	<0.001
	Session 2 × sham				
	Session 2 × cathodal L PFC vs	RT	−0.11	0.05	0.031
	Session 2 × sham				
	Stimulation × Trial					<0.001
	Cathodal L PFC vs. Sham	Chunk	<0.01	<0.01	0.037	
	Session × Stimulation × Trial					0.001
	Session 1 × anodal L PFC vs	Chunk			
	Session 1 × Sham			0.01	<0.01	<0.001
	Session 1 × cathodal L PFC vs	Chunk	0.01	<0.01	<0.001
	Session 1 × Sham				
	Session 2 × anodal L PFC vs	Chunk	<−0.01	<0.01	<0.001
	Session 2 × Sham				
	Session 2 × anodal L PFC vs	Chunk	<−0.01	<0.01	0.01
	Session 2 × cathodal L PFC				
Experiment 3 follow-up	Stimulation × Trial					0.001
	M1 vs. Sham	RT	−0.12	0.06	0.045
	M1 vs. Left PFC	RT	−0.19	0.06	0.003

*The first column describes the study, the second column describes the interaction, the third column describes the data type, the fourth column provides the beta values (β) relative to sham, the fifth column is standard error (Std. error), the sixth column is p-value, and the seventh column is the partial eta-squared (η^2^). Left (L), primary motor cortex (M1), prefrontal cortex (PFC), right (R), reaction time (RT)*.

To evaluate whether starting reaction time was associated with the learning effects (i.e., some participants might have been closer to floor or ceiling performance at the outset), we ran the same analyses with reaction time normalized to that of the first trial. This did not change the results (see Supplementary Material).

### Contrasts on Reaction Time

Hypothesis-driven contrasts revealed no differences between the stimulation groups and sham within each session.

### Offline Gains

Planned contrasts revealed tDCS to M1 did not significantly modify offline gains in reaction time regardless of the session. The lack of offline gains in the M1 tDCS group suggests that stimulation over M1 did not affect consolidation (session one vs. session two; session two vs. session three) in the *DSP* task.

### Errors

Friedman’s test revealed that there was a significant change in the number of errors across the three sessions ($\chi_{\left(2,N=62\right)}^{2}$ = 82.83, *p* < 0.001). There was a significant decrease between sessions one (median = 8, or 3% of all trials) and two (median = 7, or 2% of all trials; *Z* = −2.18, *p* = 0.029), and a significant decrease between sessions two and three (median = 1.5, or 2% of all trials; *Z* = −6.54, *p* < 0.001) in the number of errors made. There was no difference between the number of errors in the stimulation groups. Thus, learning was demonstrated across all groups reflected in fewer errors during session two relative to session one.

### Chunks

**Figure 3:**
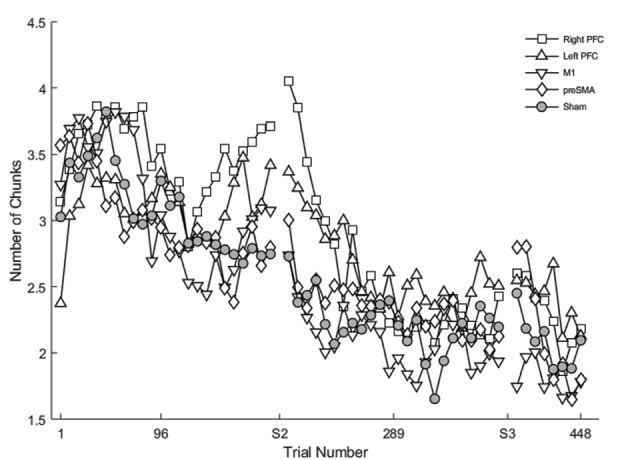
The mean number of chunks estimated by the model in the complex sequence as a function of trial number. Displayed means were binned across every eight trials. S2 and S3 on the x-axis represent the start of session two and session three, respectively.

Learning was evident as a decrease in the number of motor chunks across the 3 days of practice (Figure 3). The number of chunks across all trials within the second session decreased at a faster rate relative to the first session (*β* <−0.01, *SE* < 0.01, *p* < 0.001), and the number of chunks in the third session decreased at a faster rate relative to the second session (*β* <−0.01, *SE* < 0.01, *p* = 0.001).

Regardless of the session, both the SMA complex (*β* <− 0.01, *SE* < 0.01, *p* < 0.001) and the right PFC tDCS groups (*β* <−0.01, *SE* < 0.01, *p* < 0.001) reduced the number of chunks at a significantly faster rate relative to sham across all trials.

Within session one, across all trials, the left PFC group (*β* <0.01, *SE* < 0.01, *p* < 0.001; Figure 3) reduced the number of chunks at a significantly slower rate relative to the sham group, whereas in the second session, the right PFC group (*β* < −0.01, *SE* < 0.01, *p* < 0.001) and the left PFC group (*β* <−0.01, *SE* < 0.01, *p* < 0.001) reduced the number of chunks across trials at a significantly faster rate relative to sham. These findings complement the findings of the reaction time data which showed an initial impairment during session one and then a “catch-up” effect during session two.

In contrast, stimulation to M1 (*β* <−0.01, *SE* < 0.01, *p* < 0.001; Figure 3) and the SMA complex (*β* <−0.01, *SE* < 0.01, *p* < 0.001 resulted in a significantly faster rate of reduction in the number of chunks relative to sham within the first session. In the third session, stimulation to the SMA complex (*β* = −0.02, *SE* < 0.01, *p* < 0.001) and the right PFC (*β* = −0.01, *SE* < 0.01, *p* = 0.001; Figure 3) resulted in a faster rate of reduction in the number of chunks relative to sham. The linear mixed model applied to the simple sequences revealed a complementary pattern of results (see Supplementary Material). Thus, stimulation to either the M1 or the SMA complex facilitated chunking in sessions one and three, respectively.

### Contrasts on Chunks

Planned contrasts revealed no significant differences between the real stimulation groups and sham within each session.

### Testing Conditions

A mixed, three-way ANOVA was used to determine any differences between sessions (within), testing condition (within) and stimulation group (between). The two (session: two, three) by four (testing condition: single stimulus, familiar, mixed familiar, unfamiliar) repeated measures ANOVA was performed on the mean reaction time for each testing condition with the location of stimulation as the between-subject factor. There was a main effect of session (*F*_(1,57)_ = 47.25, *p* < 0.001), and a main effect of testing condition (*F*_(3,171)_ = 1265.19, *p* < 0.001). Reaction time in session two (*M* = 299 ms, SD ± 133) was longer than session three (*M* = 280 ms, SD ± 126). The reaction times in the single stimulus condition (*M* = 181 ms, SD ± 78) were longer than in the familiar condition (*M* = 171 ms, SD ± 66; *t*_(123)_ = 2.95, *p* = 0.004; Figure 2), and reaction times in the mixed familiar condition (*M* = 399 ms, SD ± 54) were shorter than the reaction times in the unfamiliar condition (*M* = 412 ms, SD ± 51; *t*_(123)_ = −4.72, *p* < 0.001; Figure 2). There were no significant effects of the tDCS group nor any interactions.

In summary, linear mixed model analyses demonstrated that the reaction time for the left PFC tDCS group decreased at a significantly slower rate during session one, but at a faster rate during session two relative to the sham condition. Thus, stimulation to either the right or left PFC slowed sequence production relative to sham, opposite of what we had hypothesized. The SMA complex tDCS group had significantly shorter reaction times during sessions one and two, whereas the M1 tDCS group had significantly shorter reaction times limited to session two. Stimulation to left PFC resulted in a reduction in the number of motor chunks at a slower rate during session one, but a faster rate during session two than in the sham group, whereas the right PFC group reduced chunks at a faster rate in sessions two and three. Thus, stimulation to either the left or right PFC harmed learning reflected by a higher number of chunks. These findings suggest that exciting the PFC *via* anodal tDCS may interfere with sequence learning, consistent with our findings in reaction time. Stimulation to M1 resulted in a faster reduction of the number of chunks in session one and fewer chunks for sessions two and three, whereas stimulation to the SMA complex resulted in a faster reduction in the number of chunks but a higher number of chunks in session two.

## Experiment 2

In Experiment 1, stimulation to either the right or left PFC impaired sequence learning revealed by longer reaction times and a higher number of chunks, suggesting that stimulation to either the left or right PFC impairs sequence learning. These findings support existing evidence that activating the prefrontal cortices interfere with motor learning (Galea et al., 2010; Zhu et al., 2015). To further test this interpretation, we recruited an additional 13 participants who received cathodal tDCS over the left PFC while practicing the same *DSP* task of Experiment 1 over the course of 3 days. tDCS is thought to work in a polarity specific manner (Nitsche and Paulus, 2000; Stagg et al., 2009); anodal stimulation has been shown to increase the rate of learning whereas cathodal stimulation has been shown to decrease the rate of learning in an explicit sequence learning task (Stagg et al., 2011). If anodal tDCS over the prefrontal cortices interferes with learning because the PFC contribution reduces less with practice, and cathodal and anodal tDCS are presumed to have opposite behavioral effects, cathodal stimulation over the left PFC should enhance learning. Consequently, we hypothesized that cathodal stimulation over the left PFC would result in a faster rate of learning relative to the sham group. We also expected that the cathodal left prefrontal tDCS group would learn at a faster rate relative to the anodal left prefrontal tDCS group.

## Experiment 2 Methods

We used the same design as in the first experiment with a few exceptions. The polarity of the left PFC tDCS montage was reversed, with the cathode placed over F3 and the anode placed over the contralateral orbit. Thirteen individuals (mean age 21.8 years, four males) participated. Two of the 13 participants only completed the first session of practice. Participants did not complete the MOCA, Purdue pegboard, or visual search tasks; they only completed the digit span task. We used the results of the same 24 participants from the first experiment for the anode left PFC and sham tDCS groups for comparison.

## Experiment 2 Results and Discussion

### Reaction Time

Hypothesis-driven pairwise comparisons in the linear mixed model demonstrated that the rate of change in reaction time in session two was significantly slower relative to the rate of change in session one (*β* = 0.82, *SE* = 0.03, *p* < 0.001). The rate of change in the reaction time in session three was significantly faster than the rate of change in session two (*β* = −0.31, *SE* = 0.11, *p* = 0.004).

**Figure 4:**
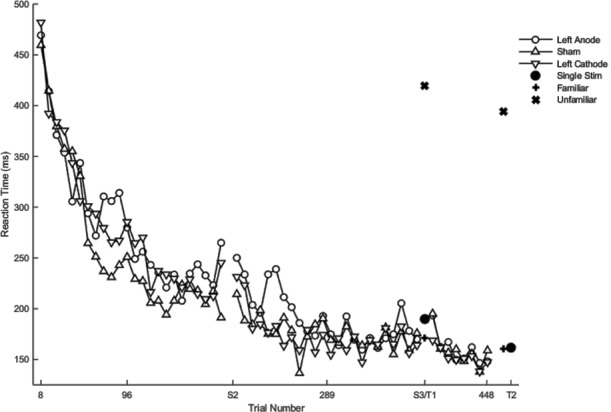
Mean of reaction time (ms) of the complex sequence as a function of trial number. Displayed means were binned every eight trials. S2, S3, T1, and T2 on the x-axis represent the start of session two, session three, testing conditions at the end of sessions two and three, respectively.

Hypothesis-driven pairwise comparisons for the first session revealed that anodal stimulation to left PFC produced a significantly slower rate of change in reaction time (*β* = 0.16, *SE* = 0.05, *p* = 0.001; Figure 4) relative to sham (results previously reported). Anodal stimulation to left PFC during session two affected the rate of change in reaction time such that it was significantly faster (*β* = −0.21, 0.05, *p* < 0.001) relative to sham. Similarly, cathodal stimulation to left PFC produced significantly faster changes in reaction time (*β* = −0.11, 0.05, *p* = 0.031; Figure 4) relative to sham in session two. Two follow-up contrasts were performed between the left PFC cathodal group and left PFC anodal group to determine whether the stimulation groups differed from each other during sessions two and three. The contrast between the left PFC anode and the left cathode in session two was significant, with the left PFC anodal group changing the rate of reaction time significantly faster than the left PFC cathodal group (*β* = −0.10, 0.05, *p* = 0.041; Figure 4; Table 2). This finding indicates that although both anodal and cathodal stimulation to PFC facilitated learning during session two, they were not identical. There were no significant findings for the third session. Thus, regardless of the polarity of stimulation, tDCS to the left PFC resulted in impaired learning as reflected by a slower rate of change in reaction time in session 1, followed by a “catch up” in session two.

### Contrasts for Reaction Time

Contrasts revealed no significant differences between any of the real and sham tDCS groups.

### Errors

Friedman’s test revealed that there was a significant difference in error rates across the three training sessions ($\chi_{\left(2\right)}^{2}$ = 53.30, *p* < 0.001). A Wilcoxon signed-rank test revealed that more errors were made during session one (median = 9.0, or 3% of all trials) compared to session two (median = 6.5, or 2% of all trials; *Z* = −2.40, *p* = 0.02) and more errors were committed during session two compared to session three (median = 1.0, or 1% of all trials; *Z* = −5.12, *p* < 0.01). It should be noted that participants only practiced the sequence for two blocks during session three. There were no significant differences between the two real tDCS groups and sham. Thus, sequence learning was reflected by a reduction in the number of errors from session one to session two.

### Chunks

**Figure 5:**
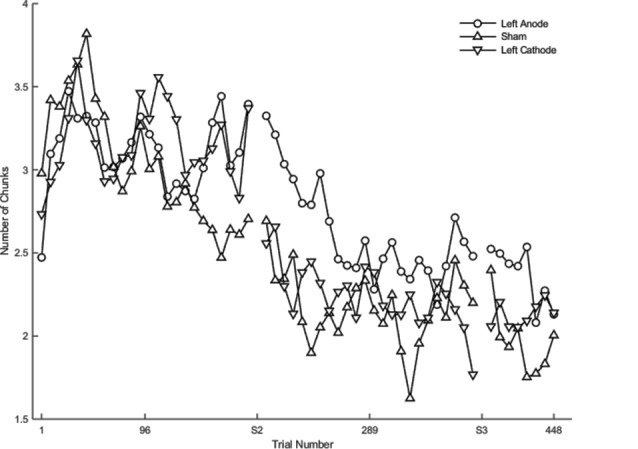
The mean number of chunks estimated by the model in the complex sequence as a function of trial for complex sequences. Displayed means were binned every 8 trials. S2 and S3 on the x-axis represent the start of session two and session three, respectively.

Regardless of the session, cathodal left PFC stimulation resulted in a significantly slower rate of change relative to sham (*β* < 0.01, *SE* < 0.01, *p* = 0.037) in terms of the number of chunks. In the full statistical model accounting for session and stimulation, anodal (*β* = 0.01, *SE* < 0.01, *p* < 0.001) as well as cathodal (*β* = 0.01, *SE* < 0.01, *p* < 0.001; Figure 5) stimulation to left PFC significantly slowed the decrease in the number of chunks relative to sham in session one. In session two, anodal stimulation to left PFC significantly reduced the number of chunks over trials at a faster rate relative to sham (*β* <−0.01 *SE* < 0.01, *p* < 0.001; Figure 5). Pairwise comparisons between the two PFC stimulation groups revealed a significant difference limited to the second session. The cathodal group reduced the number of chunks at a significantly slower rate relative to the anodal group (*β* <−0.01, *SE* < 0.01, *p* = 0.01; Figure 5). Thus, regardless of the polarity of stimulation, tDCS to left PFC led to a slower decrease in the number of chunks during session one, indicating that either exciting or inhibiting the left PFC impairs sequence learning. However, the anodal left PFC tDCS group, but not the cathodal left PFC tDCS group, exhibited a faster decrease in the number of chunks in session two. Further, the anodal tDCS group showed a benefit over cathodal stimulation in the second session. Thus, while cathodal and anodal stimulation both impaired learning reflected by the slower chunking rates in session one, cathodal stimulation resulted in a greater impairment, demonstrating no benefit in session two and a slower rate when compared to anodal.

### Contrasts for Chunks

There were no significant differences between the real tDCS groups (left PFC anodal, left PFC cathodal) and sham within each session for the complex sequences. Although both real tDCS groups consistently showed a slower rate of change in the number of chunks indicating poor learning, there were three key differences between the anodal and cathodal tDCS groups. First, only cathodal tDCS negatively affected the number of chunks during session two. Second, pairwise comparisons revealed that the cathodal tDCS group reduced the number of chunks at a slower rate when compared to the left PFC anodal group in session two. Three, the supplementary analysis revealed that the left PFC cathodal group improved learning relative to sham, which was limited to session three for both the rate of chunking as well as the overall number of chunks. These three differences suggest that although the two stimulation groups had an overall similar effect on the number of chunks, it is likely that they were mediated through differential networks.

### Offline Gains

Planned contrasts revealed no significant differences in offline gains between any real tDCS group and sham for both the reaction time or for the number of chunks between sessions one and two and between sessions two and three.

### Testing Conditions

A mixed two (session: two, three) by four (testing condition: single stimulus, familiar, mixed familiar, unfamiliar) repeated measures two-way ANOVA was performed on the mean reaction time for each testing condition. The repeated measures ANOVA revealed a main effect of session (*F*_(1,33)_ = 27.17, *p* < 0.001) and condition (*F*_(3,99)_ = 636.39, *p* < 0.001). Reaction times for session two (*M* = 297 ms, *SD* ± 136) were longer than for session three (*M* = 276 ms, SD ± 129). The familiar testing condition (*M* = 166 ms, SD ± 70) was faster than the single stimulation testing condition (*M* = 176 ms, SD ± 81), the mixed familiar condition (*M* = 397 ms, SD ± 55; Figure 4) and the unfamiliar condition (*M* = 407 ms, SD ± 50). There was also a significant session by testing condition interaction (*F*_(3,99)_ = 3.00, *p* = 0.034). We ran two additional repeated measures one-way ANOVAs to further break up the two-way interaction between session and testing condition. We found a significant main effect of testing condition for session two *F*_(1,105)_ = 437.55, *p* < 0.001 and for session three *F*_(3,105)_ = 760.69, *p* < 0.001. There were no main effects or interactions involving the stimulation groups.

In summary, regardless of the polarity of stimulation, tDCS over the left PFC resulted in a decreased rate of learning, overall slower reaction times, and a higher number of chunks relative to sham. However, despite the cathodal and anodal left PFC tDCS groups demonstrating overall similar results, contrasts between the two real tDCS groups revealed that cathodal tDCS resulted in greater learning deficits. These findings suggest that although the tDCS groups displayed similar results, the effects are likely mediated through differential networks.

## Experiment 3

Contrary to our hypothesis, the results from Experiment 2 suggest that regardless of the polarity of stimulation, tDCS to the left PFC impairs sequence learning. The impairment in Experiment 2 was reflected in both the reaction time as well as the chunking data throughout the three sessions. However, we do not know whether the impairments induced by anodal tDCS are long-lasting. A limited number of motor learning tDCS studies have included long-term follow-ups in order to assess retention. For example, Reis et al. (2009) observed enhanced motor skills across 5 days of training for the anodal tDCS group relative to sham, which remained significant 3 months later. Accordingly, we brought back participants from the M1, left PFC, and sham tDCS groups to assess the long-term effects of tDCS stimulation. Given that we observed a faster rate of learning when tDCS was applied over the left M1 and impaired learning when tDCS was applied over the left PFC in Experiment 1, we anticipated that the M1 group would display greater retention or less forgetting a year later, whereas the left PFC group would display less retention or more forgetting relative to sham a year later. We also anticipated the M1 group displaying faster relearning relative to both the left PFC and the sham tDCS groups.

## Methods

### Participants

Twenty-one young adult participants from three of the tDCS groups in Experiment 1 came back to the lab after an average of 1.3 years (14.5 months–16.3 months) from their last visit. Seven participants were from the left PFC anode group, five from the M1 group, and nine participants from the sham group. We opted to specifically invite the left anodal PFC tDCS group to participate in the follow-up study as the right PFC group did not demonstrate any benefit of stimulation in the initial study, while our findings for the left PFC group—although still overall suggesting a negative impact of tDCS—were somewhat more complex. The M1 group was included in this follow-up based on the benefits to reaction time and chunking observed in Experiment 1, along with previous tDCS literature demonstrating the long-term effects of stimulation to M1. The sham group was invited back as a control.

### Task Order

For participants’ fourth session, they completed a hybrid of sessions two and three from the first experiment. First, participants completed six blocks of practice (48 repetitions of each sequence) with their originally assigned sequences in the *DSP* task *without* tDCS. Similar to session two, after practice, participants completed the *DSP* questionnaire, then advanced to the testing portion of the *DSP* task (single stimulus, familiar, mixed familiar, unfamiliar). After the test portion, participants completed the card rotation task, visual search, visual array change task, digit symbol, and then completed an exit survey questionnaire.

### Data Analysis

The primary outcomes for Experiment 3 were offline reaction time gains, with reaction time, number of chunks, and number of errors as the secondary outcomes. For the offline reaction time gains, we used repeated-measures ANOVA using the stimulation group as the between-subjects factor and testing condition as the within-subjects factor. Offline learning gains were calculated by subtracting the mean of six keypresses (i.e., one sequence) from the first trial of session four from the mean of six key presses from the last trial of session three [e.g., session four (mean RT trial 1–6)–session three (mean RT trial 442–448)]. We did not correct for multiple comparisons. Chunking data is not available for this data set as the model fit failed given the limited number of trials.

## Results and Discussion

### Retention Interval

An independent samples *t*-test was performed on the time (in months) between session three and session four and revealed no significant differences between the left PFC group (14.5 months) and sham (16.3 months; *t*_(14)_ = −0.90, *p* = 0.39) or the M1 group (15.2 months) and sham (*t*_(12)_ = −0.49, *p = 0*.62).

### Errors

A Kruskal–Wallis test revealed no significant difference in the number of errors between the stimulation groups.

### Reaction Time

**Figure 6:**
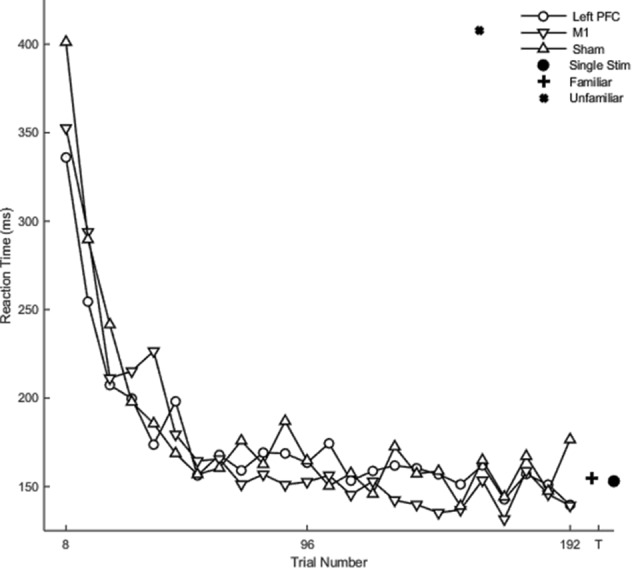
Mean reaction time as a function of trial for complex sequences for the fourth session. Displayed means were binned every eight trials. The T on the x-axis represents testing conditions reaction time.

There was no main effect of stimulation group on reaction time (*F*_(2,20)_ = 0.58, *p* = 0.56). Hypothesis-driven pairwise comparisons between the stimulation groups revealed that the M1 group reduced reaction time significantly faster during session four relative to the sham (*β* = −0.12 *SE* = 0.06, *p* = 0.045) and left PFC (*β* = −0.19 *SE* = 0.06, *p* = 0.003; Figure 6; Table 2) groups. Thus, stimulation to M1 a year earlier resulted in faster relearning of the same sequences when assessed 1 year later.

### Contrasts for Reaction Time

Contrasts between the real tDCS groups and the sham group revealed no significant differences in reaction time. Therefore, stimulation to M1 affected the *rate* of relearning, but no overall differences in reaction time.

### Reaction Time in Testing Conditions

A mixed two-way (testing condition: single stimulus, familiar, mixed familiar, unfamiliar) repeated measures ANOVA was performed on the mean reaction time for each testing condition in the fourth session. The repeated measures ANOVA revealed a main effect of condition (*F*_(3,51)_ = 515.211, *p* < 0.001). The familiar testing condition (*M* = 155 ms, SD ± 69) was significantly faster than the mixed familiar testing condition (*M* = 395 ms, SD ± 52; *t*_(20)_ = −24.38, *p* < 0.001) and the unfamiliar testing condition (*M* = 411 ms, SD ± 47; *t*_(20)_ = −25.67, *p* < 0.001). Reaction times for the mixed familiar condition were significantly shorter than the unfamiliar condition (*t*_(20)_ = 2.467, *p* = 0.023), and reaction times for the single stimulus testing condition (*M* = 153 ms, SD ± 65) were significantly shorter than for the mixed familiar (*t*_(19)_ = −29.94, *p* < 0.001) and the unfamiliar testing conditions (*t*_(19)_ = −27.80, *p* < 0.001). There were no main effects or interactions involving the stimulation groups.

### Offline Forgetting

**Figure 7:**
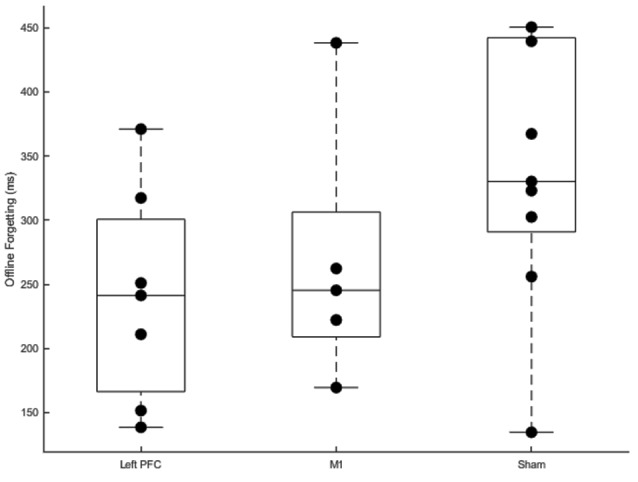
Boxplots of offline gains between sessions three and four for left prefrontal cortex (PFC), primary motor cortex M1, and sham transcranial direct current stimulation (tDCS) groups. Dots within each boxplot represent individual participants. The left PFC group exhibited significantly less forgetting than the sham group.

Offline forgetting (first trial of the fourth session—the last trial of the third session) of the complex sequences showed less forgetting for the left PFC group (Figure 7). Independent samples *t*-tests between left PFC and sham showed a near significant difference in offline forgetting (*t*_(14)_ = −2.05, *p* = 0.059) whereas there was no difference between the M1 group and sham (*t*_(12)_ = −1.25, *p* = 0.235). Thus, stimulation to left PFC a year earlier leads to less forgetting when assessed a year later on the same sequences.

Summarizing, anodal stimulation to M1 and the left PFC affected relearning and resulted in less forgetting when participants were assessed on the same sequences 1 year later. Faster relearning for the M1 group was revealed by the linear mixed model which showed a steeper slope for the M1 group relative to sham. Importantly, this faster relearning rate was not at the expense of more errors. Additionally, we found less forgetting limited to the left PFC tDCS group relative to sham. tDCS applied over the left PFC during initial sequence learning facilitated consolidation for the left PFC group a year later.

## General Discussion

In contrast with our initial hypothesis that was based on previous non-invasive brain stimulation studies (Greeley and Seidler, 2019), but in line with studies showing that prefrontal cortical activity can also interfere with aspects of sequence learning (Galea et al., 2010), we found that online stimulation to prefrontal regions impaired sequence learning and chunking in Experiment 1. In Experiment 2, we found that regardless of the polarity of stimulation, tDCS over the left PFC produced slower reaction times and slowed the decrease in the number of chunks relative to sham. In partial support of our hypothesis in Experiment 3, we found facilitation of offline gains for the left PFC group, and surprisingly no offline gains for the M1 group. However, the M1 and left PFC groups relearned their sequences at a faster rate relative to the sham group at the one-year follow-up.

### tDCS Facilitates Consolidation

Prefrontal tDCS did not facilitate sequence learning or chunk formation in Experiment 1, an effect that was consistent regardless of the sequence type (see Supplementary Material) and remained unchanged a year later in Experiment 3 when we observed that the left PFC group had offline gains (less forgetting). Together, these findings suggest that the representation of the sequences learned approximately a year earlier decayed at a slower rate for the left PFC group and remained stable, suggesting a causal role of the left PFC in long-term sequence memory. According to previous findings, the DLPFC may be involved in reordering pieces of information in working memory and subsequently enhancing memory for associations among items in long-term memory (Blumenfeld and Ranganath, 2007). More recently, Au et al. (2016) reported significant gains in verbal working memory after participants received tDCS over either left or right DLPFC. The same research team demonstrated that 12 months later, individuals who had initially received real tDCS, as opposed to sham, displayed substantial benefits to long-term retention (Katz et al., 2017). A possible mechanism underlying the long-term retention or relearning that we observed in both the left PFC and the M1 tDCS groups could involve plasticity-related protein synthesis. Plasticity-related protein synthesis was shown to be required in M1 for successful motor learning in a multi-day reaching task in non-human primates (Luft et al., 2004). Likewise, improvements in performance in a spatial working memory task in mice required the synthesis of proteins in the medial PFC, the same brain region that was active during the task (Touzani et al., 2007). These studies suggest that M1 and the medial PFC are involved in the consolidation and long-term retention of motor skills and spatial working memory strategies, respectively, potentially *via* an influence on protein synthesis. Based on this previous literature, it is possible that tDCS over either the PFC or M1 promotes long-term retention and prevents decay of motor sequences *via* protein synthesis in humans.

### tDCS Over the Prefrontal Cortices Impairs Learning

Impaired learning observed in the prefrontal tDCS groups in Experiment 1 could be due to the constant current produced by the type of non-invasive stimulation used in the present study. The *C-SMB* framework hypothesizes that the PFC prepares and initiates movement, especially once chunks have been formed. Moreover, Doyon and Benali (2005) model also proposes a time-dependent role of the prefrontal cortices which are limited to early learning, where rapid changes occur within a single session. Electroencephalogram (EEG), a neuroimaging technique that has a high temporal resolution, shows rapid changes in the brain within a single session of learning (Moisello et al., 2013; Heideman et al., 2018). Given that the brain undergoes such fast changes within a single session of practice, a constant current may interfere. Additionally, there are different types of non-invasive brain stimulation other than tDCS such as alternating or random noise stimulation which attempt to change cortical oscillations (Antal and Herrmann, 2016) rather than modulate overall activity levels. Given the specific and time-dependent role of the PFC proposed by both models of motor learning and the evidence observed in EEG studies, it may be that tDCS is too temporally crude of a technique for testing such a hypothesis. That is, in Experiment 1, modulating overall brain activity with the constant current of tDCS may have hindered the otherwise normally occurring rapid changes in the brain and subsequently impaired performance. Future studies should consider using methods that are more temporally precise and inhibit or excite the prefrontal cortices immediately before or during the first stimulus of a sequence (*C-SMB*) or limit modulation to early learning (Doyon and Benali, 2005) to determine whether this would interfere with learning or facilitate it in accordance with the two models.

It is likely that tDCS impacts the interplay between the prefrontal cortices and other cortical and subcortical structures necessary for successful motor learning. For example, the basal ganglia and thalamus are necessary for motor learning as chunking is impaired in stroke patients who had a stroke in or near the basal ganglia (Boyd et al., 2009), and individuals with thalamic lesions show deficits in visual-motor sequence learning (Exner et al., 2001). Moreover, the *C-SMB* model posits that the prefrontal cortices coordinates actions between different brain regions to successfully learn a motor sequence (Verwey et al., 2015). tDCS to prefrontal regions may affect subcortical structures such as the basal ganglia and thalamus. Symptoms of Parkinson’s disease, a neurodegenerative disease of the basal ganglia, can be transiently improved through anodal tDCS over left DLPFC (Boggio et al., 2006; Lattari et al., 2017). Further, tDCS with the anode over right PFC decreases resting blood perfusion in the right caudate in healthy young adults (Weber et al., 2014), anodal tDCS over left PFC decouples the left PFC from the thalamus (Stagg et al., 2013), and cathodal stimulation over the PFC of rodents results in an increase in striatal dopamine levels (Tanaka et al., 2013). In the context of the C-SMB model, the learning impairment observed after prefrontal stimulation may be affecting the interaction between the cognitive processor and the motor processor as activation of the cognitive processor reduces the contribution of the motor processor and ultimately leads to reduced learning. Therefore, tDCS may be impairing learning directly or indirectly *via* subcortical structures, such as the basal ganglia and thalamus.

An alternative explanation of our findings may be that PFC engagement has a direct negative impact on performance -regardless of coupling with subcortical structures. This explanation is consistent with previous literature demonstrating that engagement of the prefrontal cortices can interfere with motor sequence learning and retention, and with findings that attending to the execution of an automated skill, assumed to increase involvement of the prefrontal cortices, results in poorer performance (Beilock and Carr, 2001; Beilock et al., 2002; Gray, 2004). For example, disruption of either the left or right DLPFC with TMS immediately following sequence learning results in greater retention (Galea et al., 2010), and cathodal stimulation over the left PFC facilitates performance and retention in a golf putting task (Zhu et al., 2015). Consistent with these previous findings, we found anodal tDCS to prefrontal cortices during learning impaired motor sequence learning. Thus, the over-involvement of frontal brain regions may negatively impact motor performance. However, our results in the left PFC cathodal tDCS group in Experiment 2 are inconsistent with this idea, which may be due to methodological differences. In the Galea et al. (2010) study, TMS was used to disrupt the prefrontal cortices *after* sequences had been learned. Here, participants received stimulation *while* they learned the sequences. This particular finding is consistent with the *C-SMB* framework, which would predict keeping the PFC engaged reduces the transition from reaction to sequence mode and reduces the use of chunks.

### Anodal and Cathodal tDCS Over Left PFC

Existing current density modeling literature of tDCS including the ROAST model we used suggests that the electric field magnitude distribution across the cortex and underlying brain regions in both anodal and cathodal stimulation over left PFC are similar (Datta et al., 2012; Bai et al., 2014). This similarity may be due to the close proximity of the prefrontal electrodes and might also explain the similar behavioral results produced in this and other studies, suggesting that the canonical assumption of “anodal excitatory, cathodal inhibitory” is oversimplified (Bestmann et al., 2015). Confirming this notion, a meta-analysis by Jacobson et al. (2012) calculated that the probability of getting the “anodal excitatory, cathodal inhibitory” effect in the motor system was 0.67, where the probability for the same tDCS effect in cognitive studies was a mere 0.16. At least three other tDCS studies that have used both anodal and cathodal stimulation in the same study have found similar results regardless of the polarity of the current. Both anodal and cathodal stimulation over the cerebellum impaired performance in a working memory task (Ferrucci et al., 2008), improved semantic processing when tDCS was applied over Wernicke’s area (Brückner and Kammer, 2017) and reduced the sense of agency when applied over pre-SMA (Cavazzana et al., 2015). It may be that the learning impairment observed in the current study does not depend on a specific direction of change induced by tDCS, but rather *any* basal deviations (Javadi, 2015). Moreover, the 2 mA current used in cathodal tDCS group may have affected the brain in an identical way to the anodal tDCS groups. For example, 20 min of 2 mA of cathodal stimulation over M1 lead to an *increase* in the amplitude of motor evoked potentials, whereas 1 mA of cathodal stimulation for the same amount of time lead to a decrease in the amplitude of motor evoked potentials relative to baseline (Batsikadze et al., 2013). Given that we used 2 mA of stimulation coupled with smaller electrodes (yielding a higher current density), it is reasonable to think that we induced cortical excitability underneath the cathode instead of suppressing it. Thus, anodal and cathodal stimulation may not consistently yield opposing effects on the brain and behavior, but rather in some instances, may have similar impacts. Future studies should adopt designs which include both anodal and cathodal tDCS groups and varied task conditions to further test these assumptions.

While the behavioral results for the anodal and cathodal groups were similar, it is likely that they were mediated through different networks. Perfusion and functional connectivity studies using tDCS demonstrate differential network activation based on the polarity of stimulation. For example, anodal stimulation resulted in increased perfusion to primary sensory and paracingulate cortices and decreased coupling between the left PFC and thalami, brainstem, and cerebellum, whereas cathodal stimulation over the left PFC resulted in decreased perfusion to the thalami and decreased coupling between the left PFC and ipsilateral temporal, parietal, and occipital cortices (Stagg et al., 2013). The findings from Stagg et al. (2013) suggest that the neural underpinnings of the observed impairments in our study likely differ depending on the stimulation type. Contrasts between the two tDCS groups in our study also support this. We observed that the left PFC anodal group often produced sequences or formed chunks at a faster rate compared to the left PFC cathodal group, but overall the left PFC cathodal group often had fewer chunks compared to the left PFC anodal group. Had the cathodal and anodal tDCS affected the same brain networks in an identical manner we should have observed no differences between the two groups.

### Enhanced Chunking in the M1 and SMA Complex tDCS Groups

Expectedly, stimulation to M1 and the SMA complex accelerated chunk formation. The SMA complex and M1 tDCS group displayed a faster rate of change of chunks across all trials and session 1, respectively. These findings are in accordance with the C-SMB framework which posits that M1 and preSMA are involved in chunking. Additionally, stimulation to M1 has previously been shown to facilitate motor learning in a wide variety of explicit sequence learning tasks (Stagg and Nitsche, 2011; Saucedo Marquez et al., 2013; Waters-Metenier et al., 2014). In addition, Steele and Penhune, 2010 proposed that the striatum, responsible for motor chunking, and M1, responsible for the representation of learned sequences, work in concert to learn explicit, spatial motor sequences. Indeed, Polanía et al. (2012) have demonstrated that tDCS over left M1 modulates cortico-striatal functional connectivity. Thus, it is possible that stimulation to M1 in our study indirectly affected the striatum, thought to largely be responsible for chunking.

Alternatively, M1 tDCS could have affected chunking through the premotor cortex. In animal models, the premotor cortex has been shown to be densely connected to M1 (Godschalk et al., 1984, 1985; Fang et al., 2005). The motor learning literature in humans suggests that the premotor cortex is also engaged during chunking (Bor et al., 2003; Abe et al., 2007; Pammi et al., 2012). This is consistent with a recent finding which suggests that premotor and parietal areas are key brain regions responsible for encoding learned finger sequences, whereas the role of M1 is limited to sequence initiation *via* inputs from premotor and parietal areas (Yokoi et al., 2018). Thus, it is possible that in the present study, stimulation over M1 positively impacted M1-premotor connectivity, resulting in online gains. Given the present and previous findings, models of motor learning could be modified to incorporate the role of M1 beyond execution to chunking.

### Faster Relearning in the M1 Group

Contrary to our hypothesis, we found that M1 stimulation did not affect offline learning gains (less forgetting) or retention effects across short (days) or long (12 months) time periods. This is inconsistent with the findings of Reis et al. (2009) who found an improvement of motor learning through the enhancement of offline gains during an isometric pinch force sequence task that remained stable up to 3 months later. The enhancement of offline gains in the force sequence task but not in the current study might be due to the task-specific effects of tDCS. For example, Saucedo Marquez et al. (2013) found stimulation to M1 enhanced online gains, but not offline gains for a finger sequence learning task, whereas offline gains, but not online gains were enhanced for an isometric pinch force task. The task used in our study is similar to the finger sequence learning task employed by Saucedo Marquez et al. (2013); thus, our findings are consistent with the idea that anodal tDCS over M1 is task-specific. However, we did observe a long-term performance benefit for the M1 group as evidenced by a faster relearning rate in Experiment 3. This is in contrast with the results of the first session of Experiment 1, during which there were no differences in the rate of change in reaction time. Although, we did not observe any significant offline gains, possibly the faster relearning observed in the M1 group was mediated through consolidation. Evidence supporting this idea comes from a longitudinal, fMRI motor adaptation training study (Landi et al., 2011). In the Landi et al. (2011) study, 1 week of training led to faster relearning a year later and an increase in gray matter concentration and white matter fractional anisotropy, a metric of white matter, in the left M1. Further, greater gray matter concentration changes were positively correlated with savings observed in the same task 1 year later, suggesting the left M1 is the likely location of the stored motor representations. This is consistent with our observed findings, where we found stimulating left M1 paired with practice resulted in faster relearning a year later.

### No Long-Term Impairment in the Left PFC Group

Previous studies suggest that anodal tDCS over left prefrontal regions may transiently interfere with cognitive processes. For example, anodal tDCS to the left lateral PFC results in suboptimal decision making (Xue et al., 2012) and anodal tDCS to the left DLPFC decreases adaptive behavior (Turi et al., 2015). Similarly, we observed impaired learning in the left PFC tDCS group in our motor task, which likely engaged cognitive processes engaged in sequencing behavior (Verwey et al., 2019). However, the impaired motor learning observed in the left prefrontal tDCS group in the current study appears to be transient. During the one-year follow-up, we observed less forgetting in the left PFC group, an indication that although they initially learned less, the left PFC group also forgot less. Had the rate of forgetting in the left PFC group been similar to the sham group this would have yielded more forgetting, in addition to initially poorer motor learning. While we did not re-administer the MOCA in Experiment 3, participants showed no difference in spatial working memory capacity at the one-year follow-up (not reported). Future studies should consider the long-term cognitive impact of multiple sessions of tDCS.

### Limitations

The present study is not without limitations. We used a single-blind design with the experimenter aware of the tDCS assignment. However, the participants were poor at guessing whether or not they received stimulation. Another potential limitation is that the SMA complex and the right PFC tDCS groups were not included in Experiment 3. By doing so, we missed an opportunity to observe how two bouts of stimulation would affect forgetting and relearning. Finally, perhaps the other regions of the brain that were not directly targeted were affected by the tDCS current due to the size of the electrodes and the non-focal electric field in the brain produced by tDCS. Previous studies pairing tDCS and fMRI have found widespread BOLD activity caused by tDCS in both cortical and subcortical regions of the brain that are far from the electrodes (Peña-Gómez et al., 2012; Park et al., 2013). Thus, our results cannot be attributed to any one of the brain regions with full certainty. Future studies should consider pairing tDCS with other neuroimaging methods in order to better understand how tDCS influences the brain and behavior.

## Conclusion

In conclusion, tDCS to four different cortical regions yielded differential effects depending on the site of stimulation during the *DSP* task. Stimulation over the prefrontal cortices impaired learning in Experiment 1 but resulted in offline gains for the left PFC group a year later. M1 stimulation did not yield offline gains but did yield online gains as indicated by a reduced number of chunks in Experiment 1 and resulted in faster relearning of the same sequences 1 year after stimulation.

## Data Availability Statement

The datasets generated for this study are available on request to the corresponding author.

## Ethics Statement

The studies involving human participants were reviewed and approved by University of Michigan. The participants provided their written informed consent to participate in this study.

## Author Contributions

BG was responsible for the design, data collection, analysis, interpretation of results, and writing and preparation of the manuscript. JB was responsible for the design, the chunking analysis, and editing. WV and RS were jointly responsible for conceptualization (ideas, substance, and design) and edits.

## Conflict of Interest

The authors declare that the research was conducted in the absence of any commercial or financial relationships that could be construed as a potential conflict of interest.
