# Supplementary material for: Multi-session Transcranial Direct Current Stimulation Over Primary Motor Cortex Facilitates Sequence Learning, Chunking, and One Year Retention
Source: Front Hum Neurosci. 2020 Mar 12;14:75. doi: 10.3389/fnhum.2020.00075 (PMC7080980; doi:10.3389/fnhum.2020.00075)
Supplement: Supplementary file 1 [file Table_1.docx]

Supplementary Analyses

**Experiment 1**

*Linear Mixed Model: Reaction Time Normalized To First Trial*

To determine the interaction of starting reaction time on performance changes over time, we reran our analyses with reaction time normalized to that of the first trial. Similar to what we found on the un-normalized data, across the five tDCS stimulation groups, the linear mixed model revealed that reaction time changed faster across trials in the first session than in the second (β = -.002, SE = .0001, *p* < 0.001). Reaction time across trials in session three changed significantly faster relative to the trials in session two (β = -.0006, SE = .0001, *p* < 0.001).

We found several significant differences in the slopes of reaction time across trials within session by tDCS stimulation group. In the first session, the right PFC group (β = .0003, SE = .0001, *p* = 0.002) and the left PFC group (β = .0002, SE = .0001, *p* = 0.051) reduced reaction time more slowly across trials relative to the sham group. In the second session, stimulation to the right PFC resulted in a significantly faster rate of change in reaction time across trials relative to the sham group (β = -.0003, SE = .0001, *p* = 0.002). There were no significant interactions in the third session. Similar to the analyses performed on the un-normalized data, stimulation to left PFC and right PFC slowed sequence production during session one, which resulted in a “catch-up” effect during session two. There were no other significant differences.

*Chunks: Simple Sequence*

The simple sequences that subjects performed are designed to create a mid-sequence chunk point; they comprise a repeating triplet (e.g., CVNCVN). The analyses on these data yielded a pattern of results very similar to that seen with the complex sequences. Collapsed across all tDCS groups, the linear mixed model revealed that the number of chunks for the simple sequence decreased at a faster rate in session one compared to session two (β = -.004, SE = .003, *p* < 0.001).

Across all sessions, the SMA complex tDCS group reduced the number of chunks at a faster rate relative to the sham tDCS group across trials (β = -.003, SE = .0001, *p* = 0.016).

Regardless of the stimulation site, real stimulation slowed the reduction of the number of chunks relative to sham in session one. The right PFC (β = .003, SE = .0001, *p* < 0.001), the left PFC (β = .01, SE = .0001, *p* < 0.001), the M1 (β = .004, SE = .0001, *p* < 0.001), and the SMA complex (β = .002, SE = .001, *p* = 0.017) tDCS groups all reduced the number of chunks at a slower rate compared to sham in the first session.

In the second session, the right PFC (β = .003, SE = .0001, *p* < 0.001) and the M1 group (β = .005, SE = .0001, *p* < 0.001) reduced the number of chunks at a significantly faster rate relative to the sham group. In the third session, the SMA complex tDCS group (β = .01, SE = .004, *p* = 0.001) reduced the number of chunks at a faster rate relative to sham. All other contrasts were not significant.

*Contrasts on Chunks: Simple Sequence*

Contrasts revealed no significant differences between any of the real tDCS groups and sham.

**Experiment 2**

*Linear Mixed Model: Simple Sequence Chunks*

Across the three tDCS groups, the linear mixed model revealed that the number of chunks reduced in number faster across trials in the second session compared to the first session (β < -.01, SE < .01, *p* < 0.001). The number of chunks reduced at a faster rate across trials in session three relative to the trials in session one (β < -.01, SE < .01, *p* = 0.026).

Across all trials and regardless of session, the left PFC anodal tDCS group decreased the number of chunks at a significantly slower rate relative to sham (β < .01, SE < .01, *p* = 0.05). The left PFC cathodal tDCS group reduced the number of chunks significantly faster compared to the left PFC anodal group (β < -.01, SE < .01, *p* = 0.025).

Both the real tDCS groups reduced the number of chunks at a slower rate across trials relative to sham in session one. The left PFC anodal (β < -.01, SE < .01, *p* < 0.001) and the left PFC cathodal (β = .01, SE < .01, *p* = 0.026) tDCS groups reduced the number of chunks at a significantly slower rate relative to sham during session one. Pairwise comparisons revealed that the left PFC cathodal group reduced the number of chunks at a slower rate relative to the left PFC anodal group (β < .01, SE < .01, *p* < 0.001) during session one.

However, during session three, the left PFC cathodal tDCS group reduced the number of chunks at a significantly faster rate relative to the sham (β < -.01, SE < .01, *p* = 0.015) and the left PFC anodal (β < -.01, SE < .01, *p* = 0.004) tDCS groups.

*Contrasts on Chunks: Simple Sequences*

The left PFC cathode group (*t*(32) = 2.29, *p* = 0.034) had significantly fewer chunks compared to sham in the third session. The rest of the contrasts did not reach significance.
